# Experiences of First Aid in Air Raids

**Published:** 1941

**Authors:** 


### EXPERIENCES OF FIRST AID IN AIR RAIDS.

A MEETING of the Bristol Division of the British Medical Association was held at the Physics Department, Royal Fort, University of Bristol, on Thursday, 12th June, with Dr. J. A. Nixon, Chairman of the Division, presiding. A symposium on "Experiences of First Aid in Air Raids" was held.

In opening the meeting the CHAIRMAN said it was hoped to get together a body of experience which might be of use to those who had never had the experience of rendering first aid under raid conditions, and he asked Dr. H. M. Golding to open the symposium.

Dr. H. M. GOLDING (Mobile First Aid Unit): In another fortnight we shall mark the anniversary of the first bombing of Bristol and during that twelve months the doctors of the City have passed through many and varied experiences and as Dr. Nixon has said it seems worth while to recount some of the events and observe in what way they have affected our outlook on A.R.P.

In June last when the sirens first howled just after midnight, followed immediately by bomb explosions, the doctors at our post collected with commendable rapidity and they came in with exciting stories. One had run into debris and broken glass, another had been stopped time after time by wardens to prove his identity, a third told of great fires—exciting then, commonplace enough now, though to me the most fearsome part is still that drive alone from my home to my post in the middle of a bad "blitz." That night we had one patient with burns on the forearm and the nurses gathered round their first casualty like bees round a honey-pot.

In Bristol it has been the policy to establish first aid posts at established clinics or hospitals, although there has been some conflict between the A.R.P. staff and the peace-time staff it has had the advantage of a fully-equipped station for the first aid post. Although I am on a mobile unit I have had quite a deal of experience in first aid work, I have waited all night for casualties and caught nothing, and I have seen the post full to overflowing. As in other places the tourniquet, which we thought so important when making plans in pre-war days, has not made an appearance; on the other hand, we have met shock in all its forms and we have found that the degree of shock does not depend on the severity of the injury. A patient with a scratch may be profoundly shocked, alternatively a man was brought in who had been trapped by falling debris and he had the unusual injury of a ruptured patella ligament and a forward dislocation of the knee joint, yet he showed practically no shock at all, although to his injury was added the uncertainty of the fate of his family.

One discovery we made was how dirty patients could be. They come in with their hair full of plaster, their eyes full of dust, and their clothes and bodies simply covered in dirt. On the human side we have learnt quite a lot in our first aid post. We no longer ask a patient if he can go home until we are quite sure that he has a home to go to, we inquire whether friends or relatives can take in a bombed-out patient and if necessary we arrange for removal to a hostel. We have seen a mother and father with their boy and girl walk out of the post into the cold, strangely quiet dawn, on their way to a hostel thanking us for what we have done and comforting themselves with the thought that if all else was lost they were alive and they were together.

A mobile unit is a van fully equipped to set up an emergency first aid post near the scene of a severe incident in some convenient hall, schoolroom or other building, but unfortunately this does not work out so easily. Either there is no hall just where it is needed or else the building is not blacked out, or if it has been blacked out, the blacking out is probably blasted out and we have never been able to set up a routine post. A mobile unit is stationed at a fixed first aid post, it is sent out on orders from the Report Centre and it is manned by a doctor, a trained sister, five auxiliary nurses and a driver. My unit belied its name by remaining completely immobile until the first big "blitz" in November. We were then sent to an incident at a point I knew to be about a quarter of a mile from any suitable building in which to set up a post. A warden confirmed this and pointed out that the casualties had been collected in a large surface shelter, so we went into the shelter, half of which had been cleared to allow for a number of casualties, varying from a sprained ankle to a depressed fracture of the skull, and we had to treat them under the most difficult conditions.

I would like to speak about the other half of that shelter. It was full—men, women and children standing still in a silent patient crowd. They seemed a little scared at the sight of the wounded but were otherwise quite motionless. They watched us in the dim electric light from the roof of the shelter and suddenly there was a terrific explosion from a "near miss," the shelter filled with dust, and the light went out. The crowd gave a stifled scream and we switched on our torches and shouted out that it was only the light gone out, and again they were silent and motionless. It was with great relief we saw our casualties into the ambulances and were able to find boxes for the old people to sit on and give the others room to breathe more freely.

We have learnt quite a lot in other ways. In the *St. John's Manual* we were told that for treatment for shock we should ensure freedom from worry. I was given a sharp lesson on this: a woman and a boy were completely buried by debris, I watched them being dug out and when the woman's shoulders and head were exposed she said, "I have lost my handbag." A man standing by said, "It is all right, missis, don't you worry, the policeman has got it." Actually the handbag was dug out of the ruin some four days later, but she had been most effectively treated for shock.

Apart from work with the unit, on two occasions when I was not on duty I have been called out by wardens to attend casualties. This was before any official scheme was in operation. Once I had to go to a warden's post to see a man who was unconscious with severe head injuries and all I could do was to see he was not bleeding and get him packed off in an ambulance. Another time I went to see a woman and child who had been trapped and when they were released they were only slightly injured and shocked. I put them into the back of my car and ran them up to the post and there they had treatment. Apart from anything I was able to do for the casualties I felt my presence and advice was a great relief to the wardens and rescue men, and this is a strong reason why a doctor should be present at as many incidents as possible.

It would be ungracious of me to finish without paying a high tribute to the sisters and nurses of my unit. They have turned out in the most hazardous conditions without hesitation, they have never shirked any duty or order, and anything I have been able to do has been completed through their kindness and efficiency. Our relations with the police, the ambulance and rescue parties have always been most cordial and we have never failed to find a helpful co-operation. Bristol is having a quiet period, but if our tribulation should start again we do feel in our district that with the experience we have had we should be able to tackle any job which was given us and we should not be found wanting.

Dr. A. M. FRASER (Assistant M.O.H. Bristol): We have been asked to confine ourselves to actual personal experiences. My experience has been in Bristol's Control, where my business is to advise and help the Divisional Report Centres in the task of operating the casualty services. This term "Report Centre" does not occur in peace-time ambulance or casualty work, and I should like to say a few words on the organization of the service.

For purposes of operation Bristol is divided into six divisions, each of these is built up to be an independent unit to function by itself, which means, as regards casualty work, it has an action depot, attendant ambulances, first aid and rescue parties, at least one first aid post, one first aid mobile unit, first aid centres, an emergency mortuary and a blister-gas cleansing station. All these are co-ordinated by the officer in charge of the Divisional Report Centre. It is to the Report Centre that the Wardens, Police, and A.R.P. Services send information and make their needs for assistance known. I am stressing the existence of these centres because it happens sometimes that a member of the public needing an ambulance sends to where he sees an ambulance stationed, only to be referred to another building. The various reports that come in all have to be pieced together to give a proper picture of the incident, but having established their occurrence prompt action is essential for the work of the casualty services.

First and most important is the question of reinforcements. Each division is independent, but it is only so to the extent of its own resources, which means where ambulances are concerned about twenty and mobile units two. No one knows how big the air raid is going to be and each centre sends a copy of its action message to the Control. When the Control realizes that more help is needed than the division has at its command it makes a rough estimate of what it needs and asks Control to supply it. Some divisions may be heavily engaged and others only slightly and the latter are instructed to send assistance to the former. The necessity of having ambulances here and there makes smoother working for one district to help another rather than to call for outside help, which must be met and organized and given guides. On one occasion the regional authorities asked if we would like reinforcements. These came in during daylight and were distributed throughout the city. Had a raid occurred they would have been most useful working as part of Bristol's own organization.

The chief value of reinforcements is not during a raid night but on the following night should a repetition occur. Our system of working depots requires that men must come on duty in order that all vehicles should be manned, but if we had heavy recurring raids exhaustion would come in. I have the impression that it would be more satisfying to the county people to render first aid rather than to collect dead bodies, which was the great bulk of the work when they came in during the latter part of recent raids.

To get reinforcements by direct telephone lines is an invaluable factor. The movement of mobile units between divisions has worked well, but with the establishment of first aid centres this can be expected to work even better in the future.

The second important job is the fanning out of information from Control; we have to keep in touch with hospitals and if we find one hospital is receiving a heavy load and others not receiving at all, instructions are sent to the Report Centres to deflect ambulances to other hospitals. This does not succeed in stopping the flow entirely, but it relieves it considerably and enables patients to get quicker treatment by taking them longer distances to hospitals where surgeons and beds are waiting. We have had a case where a big receiving hospital was put out of action, a case where a depot was bombed and a mortuary bombed, and it is through Control that some other division takes up the work of the one which has been destroyed.

A third task is the calling out of mobile units; this is a feature which has not worked with any degree of satisfaction. Before raiding started it was assumed that mobile units would go to bigger incidents, wardens would report the number of casualties, but when raiding occurred these bigger incidents proved very rare and, instead, Report Centres found themselves faced with a large number of small incidents. Here also practice has corrected theory, casualties are not always obvious to the warden on his first hurried report, and it is necessary to maintain the rule of sending ambulances and first aid parties to every account of an H.E. bomb having caused damage. This means that 30 per cent. of journeys are useless, but before this rule was brought in there were many incidents of people having to wait long periods before being taken to hopsital. Wardens' reports are not such as to give Report Centres any indication of whether doctors are required, and calling of doctors was left to first aid party leaders, who were asked to send a message if they wanted a doctor. This was seldom done because they preferred to take the injured straight to hospital, they still have this power and they used it in the incident which occurred last night, but we try as far as possible to make our first aid parties and ambulances operate on the lines set down by the permanent ambulance bodies. The wardens have been asked to take a hand in calling the local doctor when they wish to do so —this scheme is in process of being hammered out. Similarly, mobile units have proved their usefulness in ways far different from those originally intended. One feature that has been noted in Control is the difficulty of contacting the doctors once a raid is in progress, and in more than one incident mobile unit doctors have rendered invaluable service by remaining immobile.

This is the main task of the Medical Section of Control and there are others such as arranging for the collection of bodies, and incidentally in Bristol we have found it necessary to use the first aid parties and ambulances largely for this work—and I shall be anxious to know if other authorities have organized some other form of service. There are cases of ordinary illness to be moved, or infectious illness to be moved from shelters and other little jobs which are necessary.

What I have said shows, I think, the nature of the work done. I would suggest that doctors knowing the telephone number of the Divisional Centre get in touch with them after an incident near them. I admit that our ambulances can seldom arrive at an incident as a peace-time ambulance would do, partly because of the clerical work and partly because an air raid plus everything else happening at the same time makes it difficult.

Dr. M. E. J. PACKER: I have had the privilege of turning out on every raid, but I am going to speak only of six incidents because of the lessons I learnt at them. The first one was in September. The two units were the first to get there and we were sent for rather late and most of the casualties had been cleared away, but there were two things which were worth mentioning.

The first was that we found a casualty who had been badly trapped in a shelter. She herself was not unconscious and my colleague got there first and he found many things were necessary for such a case, but our unit was on the road. The pre-war theory was that the unit would be set up and the doctor would not leave the building, but we were no good on the road and we had to go over grounds to get at this case. We gave her an anæsthetic and applied very strong straps round her shoulders and they pulled at her from inside while I worked from outside on one foot and with a great deal of heaving we got her out.

She was laid on the ground and I was applying dressings when another doctor arrived. This particular doctor had been the first one on the scene, he had decided that her legs were embedded and would have to be amputated. Great was her joy that we had got her out before the other person arrived with the amputation apparatus. A doctor should have emergency equipment with him when he goes to an incident. In that case we had to send back to our vans for everything that we required. After that, in Bristol, we adopted the principle of having a bag supplied to doctors so that they could have emergency outfit with them and this has proved very useful indeed.

The second matter was that we parked our ambulances as near as we could, and left our personnel with the vans. When we came back we heard there was a bomb in the road and I suggested that the vans should be moved as far as possible away from the scene ; that was done and twenty minutes after another time bomb, about which we knew nothing, blew up where our vans had been, so that the vans might have disappeared and the personnel perished. Since that time I have been on spots where there were bombs and had narrow escapes. The second point I wish to register is that we should get on to a job, do it as quickly as possible, and clear out and get any unwanted personnel away.

In the second incident the bombs fell not far from my headquarters and without waiting to be sent there I went down in my car with nurses and found a number of casualties on the site. I left one nurse in a house and told the wardens to send the casualties there, went back to the post, got the van with the nurses and went back to the incident. When I got there I found there were casualties in several houses in several more or less adjacent roads. I saw what the casualties were in the first house, left some nurses with loose dressings and went to three or four other houses. In one of them there were a couple of casualties which were not very bad, all they needed were dressings and anti-tetanic serum, but the house was so badly damaged, filth everywhere, no light, we could not give the serum and we had to send the patients back to the first aid post to have their injections. The lesson learnt from that was that our van should be so arranged that we can give A.T.S. injections in the van during the night, so as to save unnecessary journeys to the post for injections. The second lesson was that taking a van round was very cumbersome, after that I used a private car. You can take a few nurses with you, the car can be easily turned, and the van can be left at the main crater as a headquarters.

The third incident which I should like to mention was not far from here, when a Y.W.C.A. hostel was hit. I found a number of girls trapped in a basement and the rescue party were getting them out. As they came out I gave them a brief examination and sent them to a house which was opened for our use, they were supplied with hot drinks and made comfortable and three of my personnel were there to deal with the minor casualties. I went back to the main site and we could hear the cries of women who were trapped. The rescue party worked as quickly as it could and managed to get the head and one breast of one woman uncovered, there was a lot of overhanging debris, the woman was dead and we moved out. In a few moments the whole of the debris collapsed where we had been. I have a fair bit of experience of crawling through holes. I would far rather crawl through a narrow hole than a big one which is likely to collapse. I went in one place where there was a narrow hole, I wriggled through on my " tummy " and got to where another was buried, she was also dead and I wriggled back tail first. In the wriggling forward I had got my tin hat on, but afterwards I realized it was a foolish thing to do, supposing it knocked against something which released a lot of stuff I should have been buried, it would have been better to have wriggled forward without a hat. One's hair gets filthy, but that is better than being buried under tons of debris.

In the same incident I left one man to look after the woman who was buried, we came across others who were trapped, I left two nurses there and went into a further house where I climbed an improvised ladder to find a woman who had been blown under a wash-stand and was dead. It is essential for doctors to take out several nurses with them, one will not do, one helper can be left here and there and one can keep in touch with all the incidents at the same time. The night was pitch dark and I could not see who the people were or if there were people there, but when I got back I mentioned the name of my helper who would reply and there was immediate contact. The rescue party had to get to four people who were buried and had to do it by hand, when they got to the heads all that could be seen was what looked like a bit of dirty matting, the workers dug them out with a couple of fingers and thumb, it was not just loose dirt and rubble but bigger pieces. The heart-breaking part of the thing was that people asked for hot drinks, but we could not give them the drinks because there was not room to let down a feeding-cup. The rescue work was very slow and ever after that we took out a piece of rubber tubing which we could fix to the feeding cup and give them drinks that way. That is going to be all the more important now we have the Morrison shelters ; it will be hours before the people can be got out and if the rubber tube is available they can be given hot stimulating drinks.

Another important lesson was that the presence of the doctor made a difference. As soon as I arrived you could hear the whisper going round, " There's a doctor here," and it had a very good moral effect. The last lesson on that incident was that everything needed had to be fetched from the van, which involved waste of time and labour and afterwards I asked the M.O.H. if he would allow us to try a haversack, and the nurses have been wearing them since with dressings, scissors and antiseptics in them and the equipment is available immediately.

The fourth was the first " blitz " at St. Barnabas in which only a few houses were involved. As soon as I arrived on the scene, which was a little late, they said a lady must see the doctor at once. I told one of my nurses to go to see this lady and she went straightaway with her haversack. I went to where the bomb had dropped where two people were buried. There was no prospect of them being dug out yet and I left one person there as a contact, left a couple of nurses in the van and took a couple more in my car to the next road where I was told there were some casualties. By the time I got there they had been removed, two of them serious, but I had not been there of course. I must emphasize the importance of having members of the personnel at the various spots watching on your behalf while you go somewhere else. The lesson beyond this was the value of the pack and the second lesson is that the doctors should be called at the same time as the first aid parties are sent out. Doubtless other doctors have found the same thing. What happens when an F.A.P. finds a bad hæmorrhage ? He says there is going to be a lot of delay, this case must be got into hospital and rightly he sends it in; the same with a bad case of shock, he sends it to hospital, the result is the doctor rarely sees these bad cases. If the doctor was sent at the same time as the first aid party it would be worth it because all the bad cases would be seen by the doctor. The doctor need not stay to do all the dressings, he can give his instructions and leave.

The fifth incident was near my headquarters and I went there with a nurse in my car. I found there were only four casualties, two very bad, one being a bad hæmorrhage. He was bleeding profusely and a policeman was putting on an improvised tourniquet. We got them into my car and rushed them back to the post. The other case was a fractured femur with other complications, we got on a Thomas splint and rushed him to hospital, that supports my argument that doctors should go out with the first aid parties.

The last case was where we had a call to Cheriton Place ; it was entirely my mistake but I got the message, I knew Cherington Road and I shot straight off to the incident with the van following hard on my heels. I got to 24 Cherington Road and found some nasty cases there, one being a bad lung blast case. I have never seen anyone in such bad pain, he was leaping about on the ground on his back, if you understand what I mean, but the van with my case in it did not arrive. As the van was coming with me I did not trouble to go back to it to get my bag and the van did not come. I waited some little time, went back to hospital and found that the correct call was 24 Cheriton Place, not 24 Cherington Road. I had to get some morphia and send it to the nurse, but it meant a very bad hour of suffering before the morphia was given and I went on to the correct address. It impressed on my mind never to go out without my bag, and always to keep it with me and never be separated from it.

DR. J. MORTON EVANS, Jnr. : There is little that I can add to what has already been said. I have a mobile unit situated in the centre of Bristol, and our difficulties there are mainly getting from the post to the casualty. That is due to the fact that we are situated in a comparatively unhealthy spot where raids usually occur.

The first time I was asked to go out was a single case of a man with a heart attack about half-a-mile away. We started off, I took an orderly and emergency case, and walked ; and that taught me one thing, that one does need kit which can be easily carried and therefore I have three things in my pocket : I have a torch, a hypodermic in a spirit case and a rubber-capped bottle filled with a solution of morphia, $\frac{1}{4}$ gr. to the c.c. That is useful because at an incident one is not able to get sterile water and one has to work in the dark so that with a rubber-capped bottle one can push in the hypodermic, fill the syringe and put it into any part of the " lump of dirt," which is what the casualty is at the incident.

The second thing I learnt was for personal comfort one needed outside protection for one's clothes. I use a muffler, an overcoat, an old pair of golf trousers and shoes and gauntlet gloves. When I went to the case of the heart attack and on other occasions we had to get down very rapidly, the whole of the road was covered with glass and my orderly had quite bad cuts on the hands because he had not got gloves on. That is one small point which I have learned from experience.

As a mobile unit I have not functioned. At the first aid post the doctor and I work together unless the mobile unit is called out. We have had quite a few casualties and I thought possibly it might be of interest just to give you the percentages of injuries we have seen. Out of 250, 51 per cent. have been burns to the eyes or eye injuries ; $7\frac{1}{2}$ per cent. burns to the hands and limbs ; 10 per cent. head injuries, cuts, etc.; 27 per cent. wounds of the limbs ; $1\frac{1}{2}$ per cent. wounds to the trunk, and 3 per cent. shock and blast. That is the main heading under which one treats them, but in many cases, of course, you may get injuries as well as shock, but 3 per cent. were pure shock or blast. Of that number 10 per cent. were sent to hospital, half of them directly, which means that they were cases which had to receive hospital treatment urgently, and the other half were asked to attend next day for skiagram or other treatment. I do not think there is very much one can say about incidents. Indescribable dirt stands out as an obstacle every time. To attempt at the scene of an incident to try to use any form of asepsis is hopeless, one can just do the least possible amount and get the person sent elsewhere, either home (and by home I mean any neighbouring house that will take them in) or some other suitable place.

When one has seen the immediate cases and sorted them out one can go to the houses which are more or less damaged, with no hot water and nothing to help, and send the minor cases to first aid posts and the severe cases direct by ambulance to hospital. One point to which I might refer was the large percentage of burns to the eyes that we have had, that is due to the fact that we are situated in the centre of Bristol, and all the 51 per cent. have been firemen, who if they have not had burns have had smoke in their eyes to such an extent that for the time they have been completely blind, and by washing their eyes out and putting in a little liquid paraffin they have had great relief. I should like to pay a compliment to the A.F.S. in that every man went back to his job. That is all I can tell you of my impressions of the best first aid one can do under the circumstances.

Dr. R. J. STEPHEN : The first night I had to be on duty at the clinic I was down there from about a quarter to seven Sunday evening to 9 o'clock on Monday morning. The doctor should be sent for as early as possible, because that night I went down before I was sent for and incendiaries were falling in clusters.

When I got over Bristol Bridge a factory was blazing and I spent the whole night at the first aid post. I saw 62 casualties, 56 were eye burn or foreign bodies in the eyes, mainly of firemen who were led down by messengers, one man with a rather severely burned face, two cases were abrasion and cuts of the hands and several cases of burns on the forearm and several cases of hysteria, two of which were soldiers. The difficulties that night were mainly in carrying out the treatment. I do not know whether the electric light was cut off by a bomb or because of the fires, a bomb dropped so close that the treatment room was blasted in and the whole building was full of smoke and we were all filthy by the morning. The difficulty was getting a light to see the lesions of the eyes, we tried the torches from our batteries in the cars, then we tried the calor gas outfit which was carried in the mobile van, but we could not get a decent light and we resorted to hurricane lamps and treated the casualties as well as we could. I felt there was a need for some antiseptic solution to instil in the eyes, we could only wash them out with an alkaline solution and put in liquid paraffin. Several of them were in severe pain because the foreign body had got into the conjunctiva and was buried. I do not know if any of you have any comments on that side.

The second "blitz" I was at was about ten days later and it was very uncomfortable. We had 18 cases, 10 were eye burns and foreign bodies. One man was brought in for shock, there were several lacerated scalps, arms and legs. In March we had a fairly heavy raid and I went down to the first aid post early. We had 11 cases there, three of them were burns and foreign bodies in the eyes due to explosive incendiaries, a sailor with a lacerated leg and a man who died within a quarter of an hour who had the whole of one side of his face blown away and his neck exposed to the vertebral column; a fractured patella, various degrees of burns—one man came in who had been treated with bleach ointment for a burnt hand and he was in considerable pain. After that we were sent out to an incident where one of the semi-underground shelters had had a direct hit. Five people had been moved out before I got there, we had difficulty in getting down because the whole street was jammed owing to a fire. There was one casualty I saw brought out alive, another 39 were brought out dead afterwards. There was a little boy trapped by his left foot, a boy of about 6, he was quite bright and cheerful, one of the first aid workers stayed by him all the time keeping him warm and talking to him. His dead mother's head was sticking out in front of him and his younger brother's legs were visible. We got him released in a couple of hours and he was in good condition and quite cheerful. We had lacerated skulls, leg wounds, severe multiple injuries, where arms and legs were simply pulpy masses of bones and flesh and clothes. You could do nothing but lay a dressing on it, they were so badly shocked that there was no bleeding. These cases were taken into the place where we set up a post, we had to use the church, we put the stretchers on top of the pews and used the aisles and passages to do our work. The people were filthy. I had difficulty in making sure whether they were alive or dead. Some were dead and others died within five minutes, the eyes were glazed. Bombs were falling outside and in using a stethoscope I could only hear my own heart pumping. I could not feel a pulse in any of these patients. I reserved as much water as possible for hot drinks. We kept going in the church for some hours, I left the sister to attend to the minor injuries and the rest of the time I went round with various first aid people and the wardens to see whether people were dead or alive. I think I saw 18 to 20 dead people in various houses.

There was very little first aid I felt we could do on the spot; one could not suture them, they required anæsthetics and I felt that the only thing to do was to get them into hospital or a fixed first aid post. I used a vaccine bottle that I had previously made up with morphia, but one could not use more than $\frac{1}{2}$ c.c. and quite a few recovered sufficiently to be sent to hospital. Whether they got there alive I do not know, they were so badly shocked we could not even get their names and addresses.

The last occasion on which I was called out was on Good Friday when we had half-a-dozen casualties. The difficulty of working at the clinic has been when the light and water supply have been cut off for a time or because of the wreckage in various places.

Dr. W. H. HAYES: All the doctors you have heard so far have been speaking from their experience as members of the official organizations. I have just joined a rota but I am speaking as a doctor who has gone to incidents from home. It is surprising how few incidents doctors have been to because until recently there has been no organization for the average G.P.

There is no lack of willingness on the part of the doctors to go to cases and no lack of incidents, but when you think of a dark night and you are in some sort of shelter and there is a rush and a bang not very far away and you go to see you will not know where the bomb has come down; if you go to look you run the chance of collecting the next one or some shrapnel. It is not, therefore, wise to go out hunting on a "blitz" night. We are hoping that the organization will soon be completed, and there will be a doctor available for any incident which occurs without going any great distance and without having to call out the mobile units unless there is something big.

Most of what I was going to tell you has already been said. I thought I would put it in the order of happenings. There are preparations which you might like to make before you go out. Dr. Evans has told you most of the tips; gum boots are very useful, one is stumbling over all sorts of debris and broken stuff. Eye shields are useful to keep dust and smoke out. I can only endorse what Dr. Evans and Dr. Stephens have said about morphia. It is useless "fiddling" about with tablets or ampoules. I took ampoules the first time I went out but it was useless, I did not know how much dirt I injected, the whole site was covered with dust. It is impossible to put on any dressings and we should have something in the way of army field dressings which can be put straight on and bandaged round.

At the incident one finds three types: very little injured who need very little attention beyond a certain amount of reassuring; more severely injured who have to be dug out, and the dead. We get more dead than severe injuries. For the severe injuries there is little you can do, everything is filthy, give them morphia if they need it, some cases I have found do not need it, the thing to do is to get them straight to hospital. The ambulances are fairly good at turning up, although they are a bit late. I think we might have more blankets, particularly in the winter time, and possibly hot water bottles. One man we dug out after four hours with the help of straps did not appear very injured, he kept up a pretty good flow of instructions and otherwise to the people digging him out. I gave him some morphia to quieten him down, and he was very cold indeed. We put all the blankets we could find on him. Whether he managed to get warm I do not know, but he died, apparently from uræmia as a result of shock. One cannot do much other than give morphia—it is a lot—and by helping first aid parties and giving moral support.

Then there are the dead. We can be a great help. Somebody has been dug out, a part of a person is reached, and there is a cry for "Doctor." You go over and they say, "What about it ?" If you can tell them that the person is dead the next procedure is to put a rope round them and haul them out. If they are alive they have to be moved more carefully. But it is not easy, in fact I have had qualms the next day that I should be told that a case was alive when sent to the mortuary.

The stretcher party seem much happier when the doctor is there. The only other points are other types of casualties one is liable to meet. There is always the possibility of a shrapnel wound, there are more incendiary burns from the explosive incendiaries and the eyes are apt to suffer. The eyes are apt to get full of smoke and one other case, which is a matter of interest, in the very first alert we ever had I had my first casualty—I was pleased at having one at all—and it was somebody who had cut her arm in getting into her Anderson shelter.

Dr. H. D. PYKE: I am not attached to any special unit. I have been called out several times, the most important being the second "blitz." I had just come from seeing a girl who carried out an incendiary bomb in her hand and some wardens asked me to go to a major incident. The electric light had gone, fortunately I had some ampoules of morphia, I took the anæsthetic and things to amputate. The scene was very eerie, there were a large number of people underneath, the place seemed full of people in different coloured hats.

As soon as I got there I was ordered off by a policeman who said there was a doctor there but he turned out to be an attendant in a sick berth in a battleship. I was there from 12 till 5. It was a grim, dirty business. The first casualty that came out I did not know whether she was an old lady of 90 or a girl of 11, I sent her to hospital. The next was a dear old lady of 70 to whom I gave morphia three-quarters of an hour before they got her out and she was sent to hospital. The next one was dead. There was a little lull. There was a striking man there, the incident officer, he had a fine, intelligent face. He went all over the rubble and found another man alive, a little bit of stone was moving and a soldier was found, and as soon as he was free he swore like a soldier. He took a lot of getting out, we had to get some oxygen for him, he was nearly suffocated, and after that it was a question of pulling out dead people. The incident officer was very intelligent, he kept the crowd in order. The next day I was called to see him, he was crying like a baby and he has been useless ever since. The most useful people are the people who use the pickaxe and the spades. There was a crowd who grumbled hard for two hours because the next shift had not come on, the shift turned up and instead of going off the first shift stayed on and were still there when I went off.

I thought I was fairly tough, but I felt sick when I went home and for the next fortnight I felt squeamish inside. There was something dirty about the whole business, and I felt if I should ever be buried like that, and it was five hours before I was dug out, I should like to think that a doctor had some morphia which could be administered. I feel it is not right that first aid people should not have the moral support of the doctor. When I get home I often feel I have done nothing except give moral support. But if I were a victim I should like to feel there was a doctor handy and that he had the equipment to use. When I heard that there was a movement that people who were trapped should have medical help, I supported it, and it seems to me it is very slow going through. I should like to feel that there was some very well worked-out scheme where there would be help forthcoming.

Dr. I. G. DAVIES: I have no first-hand first aid experience at all, I have only treated two minor injuries in the whole of the raids, but there are one or two things I should like to say. It seems to me that some of our problems and difficulties have started from misconceptions as to the nature and type of the attack we were going to have.

I remember in the early days in 1935 or 1936, when this got going, asking somebody just what would an air raid be like and I was told "We shall hear the siren go, and about fifty German planes will come tearing across the city, there will be an unholy noise for about ten minutes, it will all be over, the all-clear will go and we shall be able to get to work."

That idea went quite quickly, but that is what I was told and I think a lot of our difficulties arose from that and have arisen from the annoying habit the Germans have of attacking in waves. The idea was that we should have mobile units staffed by doctors and nurses, they would go to the scene of the incident, set up an advanced dressing station and get down to work. But that does not happen. In Bristol itself only three times have we ever approached that state of affairs, and the direction in which the units appear to be moving now is a kind of mobile stores from which doctors and nurses go out with an emergency kit to the various scattered incidents. It is difficult to set up an advanced dressing station with bombs falling, lights and water failing and the dirt which is all round. We have made doctors much more mobile and I have been engaged in getting doctors to the spot without all the mass of equipment which was thought necessary.

The next misconception was that there were going to be large numbers of casualties and not so much damage to property. We visualized masses of people being mown down in the streets and did not seem to realize that they would have to be dug out, which led to a difficulty about the composition of the first aid parties and rescue parties. We thought in two compartments ; we thought of first aid party men who were going to do a job of first aid on the streets and we were going to have mobile dressing stations with the badly injured going to hospital. Rescue parties were going to dig people out from damaged houses. That is not what happens at all. People do not get mown down in the streets, they have to be dug out of houses in dust and in an awful mess and that is the direction in which the first aid parties are moving. They must know how to dig people out of wreckage and rescue parties must know the best way to get them out. In the early days there was a party known as the emergency party, which was a mixed party of first aid and rescue workers, and I am not sure that that is not a very good thing. My own feeling is that I would like to see the ambulances and first aid parties much more combined than they are now. I regard the ambulances as being merely vehicles plus a trained crew. We should combine the first aid parties and the rescue parties, each knowing the other's job, with a small crew to see the people safely to where they are going.

Another lesson we have learnt is the question of reinforcements. They must come early. On two occasions I have asked for reinforcements and they have had to come a long way. The decisions should be made in the beginning because otherwise they have nothing more to do than to collect up the dead.

The next thing is the necessity for an intelligent incident officer. We have not been very successful in Bristol, but there must be somebody who does take charge of the whole incident. It is difficult to find such a man but he should be found and should be in charge.

Lastly, in one raid we took samples of figures—they are not statistical samples—to give us some sort of indication. 76 per cent. of the killed in this raid were killed by splinters, 8 per cent. by blast—by which I mean there was no trace of injury to the body externally—and 16 per cent. were killed by crushing or falling masonry. Of the injured we found 38 per cent. of cases injured by splinters, 8.8 per cent. by blast, and 52 per cent. were seriously injured by crushing and falling masonry. We did an analysis of people injured in open streets, some of the impressions which came from the figures were that 52 per cent. were injured by crushing or falling masonry and only 16 per cent. were killed by the same cause. This suggests that as a large number of injuries were not severe enough to cause death, a substantial proportion might have been prevented by an efficient indoor shelter sufficient to bear the weight of the falling material. 42 per cent. were injured by damage to private houses, while 16 per cent. were killed by the same thing, which seems to bear out the idea that an indoor shelter would save a large number of casualties. This was before the Morrison shelter was delivered and I am told in Merseyside that that has happened. One of the essential features is that the warden must know where the shelter is, it should be away from the wall so that the rescue parties are able to go straight there and get the people out quite quickly. For that particular raid I tried to draw up some risk figures : killed, males 1·3 per cent., females 0·9 per cent., children 0·4, and the risk of serious injury males 1·8, females 0·7 and children 0·3, which means that men run nearly double the risk of women and over three times the risk of children of being killed and nearly three times the risk of women and six times the risk of children of being seriously injured. This is a rough sample which we were able to take for that particular raid.

Dr. N. S. B. VINTER : There have been many interesting points raised. The area in which I work borders on Bristol. We are organized under the Gloucestershire scheme, but we work closely with Bristol though there are one or two different arrangements. We are a small enough area to have been able to call a meeting of all our doctors and draw up an entirely unofficial scheme, but we have hopes of its working. We drew up a rota including all the names of doctors in the area and they were asked to allow their names to be placed on this rota and to be available for call if they were needed. That scheme has not been called on yet.

In the early raids it happened that I was on duty on two successive bad nights. The first aid mobile posts are not on the spot, they are given forty minutes to get on the road. On the bad nights I went to the post on my own initiative and went out with the first aid parties to assist in removal from an area where there was an unexploded bomb and I think they were glad of my assistance. The presence of a doctor is of great assistance to the first aid party members when they are working. In the area the working of the ambulance cars with the aid parties is as close as has been desired by the last speaker. They do work closely together, I have never seen a party go out without an ambulance ; they work from the same depot. The mobile posts have never been called out nor have we had to call the personnel together. The personnel live at various points and have to be collected by car on receipt of a warning that they might be wanted.

Col. E. T. POTTS, of the Ministry of Health : I came here to-day to listen, not to speak. One thing which has impressed me is that many of the speakers are medical officers in charge of mobile units and they almost all say how infrequently they have been called out to incidents. Mobile units were originally started for work in rural areas and not for work in cities, yet experience has shown in some large cities that mobile units can be of very great value.

In one or two big raids in London they have been called out and used very frequently and have done very good work, and in Scotland too. On Clydeside they were used on several occasions and also did valuable work, but, of course, it is no use calling out a big unit like a mobile unit in its completeness to a small incident of three or four casualties. You must have something like twenty or thirty to make it worth while calling out a unit of that kind.

One of a mobile unit's greatest uses has been the use of the doctor and a portion of the staff as incident doctors and that has been mentioned by nearly all the speakers—the importance of a doctor with a nurse or orderlies getting to an incident at the earliest possible moment, and that is what the Ministry is trying to attain now. This may be done in several ways. It is possible, in addition to the medical officer in charge of the mobile units, to have a rota of doctors who will be prepared to go to incidents—scattered over the town so that there will be somebody available nearby where the incident may occur. I rather wished that a speaker from Cardiff or Wales had spoken on this point because they have developed a scheme which is very sound and in many other places too they have this rota of doctors who can be called out from Control, and it has been provided that a warden may call out a doctor direct to an incident. That is a very good plan but it wants one proviso, namely, that if a warden calls out a doctor he must advise Control that he has done so, and with that proviso the scheme should work very well indeed.

One other point which impresses me is the question of the administration of morphia at incidents. That is of the greatest importance and as regards the method of administration, the original issue was in the form of tablets. It was brought to the Ministry's notice very strongly how difficult it was to dissolve the tablet in the dark and put the solution into the syringe and inject it. It has been decided that all first aid posts and mobile units will be issued with liquid morphia in rubber-capped bottles so that it can be conveniently used in the dark. That is supplied now and if there are any first aid posts or mobile units who have not got them, they should apply for them as quickly as possible.

One speaker mentioned the types of injuries which one sees. The figures he gave corresponded very well with the figures that I have to keep of the types of injury that come to the Ministry from first aid posts all over the country, and one thing that sticks out from the curves I make is that for every month since last September the highest figures are for injuries to the head and face. That has not varied since September ; the second highest is wounds of the upper extremity, then you drop down to wounds of the lower extremity and so on. Burns are increasing and have increased quite appreciably since the use of incendiary bombs in such large quantities. One other interesting point arises and that is that the cases of shock, not associated with injury, are decreasing in spite of the increase in the intensity of air raids. I have not seen the figures for the month of April, but that has been our experience during the last few months.

DR. TREVOR JONES (Cardiff) : Cardiff has taken all the doctors who are in active practice and who are willing to do the work and has attached them to individual wardens' posts. There is a close association between the posts and the doctors who live near and know the warden personnel and are often the family doctors of the wardens and the people around.

When an incident occurs the warden sends immediately for the incident doctor who goes without any delay of messages through Control. He is supplied with a bag containing the requisites which have been mentioned by previous speakers, notably liquid morphia and syringe. In this bag he has other items which may be necessary. Immediately he is called out a message is sent to Control saying that he has been sent out. The Control knows then whether the doctors are available. There are not enough doctors to go round all the wardens' posts and sometimes they are attached to more than one post. We have had senior medical students from the Medical School available for the same cause. There has been a close parallel in our experience with what has been described in Bristol and our experiences have been very similar. The opportunities for the use of mobile units has been very scarce.

The CHAIRMAN : We owe a debt of gratitude to all those speakers who have given their experiences, particularly Colonel Potts and Dr. Trevor Jones and several other Cardiff doctors who have taken the trouble to come over here. I think that this meeting has been a success, and it is in my mind to have a similar symposium regarding the casualty services in the hospitals on the victims of air raids so that we have some knowledge of the surgery needed for air raid victims. If I have the least support I shall try to get a similar symposium on a convenient date in this place.

The audience signified its approval of the Chairman's suggestion, and it was agreed that he should make the necessary arrangements.

